# Dynamics of Infection of Atypical Porcine Pestivirus in Commercial Pigs from Birth to Market: A Longitudinal Study

**DOI:** 10.3390/v15081767

**Published:** 2023-08-18

**Authors:** Alexandra C. Buckley, Juan-Carlos Mora-Díaz, Ronaldo L. Magtoto, Amberly Van Hulzen, Franco Matias Ferreyra, Shollie M. Falkenberg, Luis G. Giménez-Lirola, Bailey L. Arruda

**Affiliations:** 1 Virus and Prion Research Unit, National Animal Disease Center, Agricultural Research Service, U.S. Department of Agriculture, Ames, IA 50010, USA; 2 Department of Veterinary Diagnostic and Production Animal Medicine, Iowa State University, Ames, IA 50011, USA; juanmora@iastate.edu (J.-C.M.-D.); rmagtoto@iastate.edu (R.L.M.); jergens@iastate.edu (A.V.H.); francomf@vet.k-state.edu (F.M.F.); luisggl@iastate.edu (L.G.G.-L.); 3 Kansas State Veterinary Diagnostic Laboratory, Kansas State University, Manhattan, KS 66506, USA; 4 Ruminant Disease and Immunology Research Unit, National Animal Disease Center, U.S. Department of Agriculture, Agricultural Research Service, Ames, IA 50010, USA; smf0076@auburn.edu; 5 Department of Pathobiology, College of Veterinary Medicine, Auburn University, Auburn, AL 36849, USA

**Keywords:** atypical porcine pestivirus, APPV, congenital tremors, longitudinal study

## Abstract

Atypical porcine pestivirus (APPV) was found to be associated with pigs demonstrating congenital tremors (CT), and clinical signs in pigs have been reproduced after experimental challenge. Subsequently, APPV has been identified in both symptomatic and asymptomatic swine of all ages globally. The objective of this research was to perform a longitudinal study following two cohorts of pigs, those born in litters with pigs exhibiting CT and those born in litters without CT, to analyze the virus and antibody dynamics of APPV infection in serum from birth to market. There was a wide range in the percentage of affected pigs (8–75%) within CT-positive litters. After co-mingling with CT-positive litters at weaning, pigs from CT-negative litters developed viremia that was cleared after approximately 2 months, with the majority seroconverting by the end of the study. In contrast, a greater percentage of pigs exhibiting CT remained PCR positive throughout the growing phase, with less than one-third of these animals seroconverting. APPV RNA was present in multiple tissues from pigs in both groups at the time of marketing. This study improved our understanding of the infection dynamics of APPV in swine and the impact that the immune status and timing of infection have on the persistence of APPV in serum and tissues.

## 1. Introduction

Congenital tremor (CT) in pigs is a neurologic disorder characterized by tremors of the head and body that, depending on the severity, can have a negative impact on the ability of piglets to suckle [1]. This disorder has generally been classified into two types based on histologic lesions, with Type A associated with hypomyelination of the brain and spinal cord and Type B presenting no lesions [2]. Within Type A, there have been several causative factors identified, including classical-swine-fever viral (CSFV) infection (A-I), an unknown infectious agent (A-II), inherited genetic defects (A-III, A-IV), and toxicosis (A-V) [3]. Through sequencing efforts directed at affected animals, a novel virus, atypical porcine pestivirus (APPV), was identified as a possible causative agent for Type A-II CT, and subsequent challenge studies with pregnant gilts resulted in litters with CT-positive piglets [1,4,5].

After the discovery of APPV, testing of swine populations revealed the widespread distribution of this virus with positive animals found across the Americas, Europe, and Asia [6,7,8]. Globally, studies demonstrated considerable genetic variability not only between countries but also within countries [9]. Now classified as species *Pestivirus K* of the genus *Pestivirus* belonging to the family *Flaviviridae*, APPV is an enveloped, single-stranded, positive-sense RNA virus that encodes a single polyprotein that is processed into twelve mature proteins (N^pro^, C, E^rns^, E1, E2, P7, NS2, NS3, NS4a, NS4b, NS5a, and NS5b) [5]. Enzyme-linked immunosorbent assays (ELISAs) have been developed against both structural (E^rns^ and E2) and nonstructural (NS3) proteins [10,11,12]. Difficulties growing the virus in cell culture has limited research efforts with APPV, but recently, some groups have had success and have been able to perform a virus neutralization assay that demonstrated correlation with E2 ELISA [8,11,13].

Atypical porcine pestivirus can be detected in serum, feces, and oral fluids for an extended period in some animals [1,11,14,15]. In addition, tissues from pigs collected months after the cessation of clinical tremors demonstrated viral persistence in the cerebellum and lymph nodes [16]. Other swine pestiviruses, particularly Bungowannah virus and CSFV, have demonstrated the ability to cause persistent infections [17,18]. The timing of infection, especially in dams during gestation, plays a significant role in the immune response of infected animals and the potential to generate immunotolerant offspring for CSFV as well as other economically important pestiviruses, including bovine-viral-diarrhea virus (BVDV) and border disease virus [19].

Although several cross-sectional studies have been performed that have shed light on the prevalence of APPV in different swine populations around the world, longitudinal studies characterizing APPV infection over time are limited. Those that have been published have followed a small number of animals [1,11,14,15]. The objective of the current study was to further characterize the viral dynamics of APPV and host humoral immune response, using both an E^rns^ and NS3 ELISA, in a large cohort of commercial swine (n = 97) and have monthly serum samples collected from birth to market from pigs born in both CT-positive litters and CT-negative litters.

## 2. Materials and Methods

### 2.1. Animal Care and Use

This study was performed under field conditions in which animals were bled once a month under the guidance of a herd veterinarian. Animals were housed according to swine-industry standards and were humanely cared for.

### 2.2. Longitudinal Field Study

A commercial sow farm located in the Midwest of the United States experiencing an outbreak of CT in pigs was identified for longitudinal sampling. Five litters containing at least one pig with CT were enrolled in the longitudinal study, and three litters without CT were matched based on parity of the dam and age of pigs upon study enrollment (Table 1). Four out of five CT-positive litters and two of the three CT-negative litters were farrowed by gilts, and pigs were approximately two weeks of age at the first sampling. The remaining CT-positive litter and CT-negative litter were third-parity sows, and pigs were 3–4 days old at the time of the first sample collection. Litter sizes ranged from 11–13 pigs. There were 60 pigs in CT-positive litters and 37 pigs from CT-negative litters, for a total of 97 pigs.

Once a month, enrolled animals were bled for serum collection and observed for clinical signs of tremors. The first sampling and observation occurred while pigs were housed by litter with the dam, and all animals were given an ear tag for identification. The remainder of the monthly sample timepoints were collected at a wean-to-finish barn where pigs from CT-positive and CT-negative litters were co-mingled in pens (n = 6 timepoints total). Due to the nature of field studies, some animals were unable to be sampled on all timepoints due to death and ear tag loss. Blood samples were collected from the jugular furrow into serum separator tubes, and serum was aliquoted and frozen at −80 °C for future testing. Observations for visible tremors were recorded as yes or no, and an animal was classified as having CT if the animal was recorded as yes at any timepoint in the study.

When pigs were ready for market (~6 months of age), four pigs that demonstrated tremors and four pigs from CT-negative litters were necropsied for tissue collection to evaluate viral distribution. Tissue selection was based on published reports that tissues with the highest levels of APPV nucleic acid tended to be associated with central-nervous-system (CNS) and immune tissues [20,21,22,23]. Tissue samples included cerebellum, tonsil, mandibular salivary gland, palatoglossal arch, and mandibular lymph node, which were frozen at −80 °C for APPV RNA via real-time RT-PCR (RT-qPCR) quantification, and a section was placed in 10% neutral buffered formalin for RNAscope in situ hybridization.

### 2.3. APPV Nucleic Acid Quantification

Tissues were thawed and resuspended in 1–2 mL of TRI-Reagent^®^ (Life Technologies, Carlsbad, CA, USA) in individual gentle MACS™ M tubes (Miltenyi Biotec, Bergisch Gladbach, Germany). Tissues were dissociated using a gentle MACS™ Octo-Dissociator (Miltenyi Biotec) following the manufacturer’s recommendations. APPV RNA was extracted from tissue homogenate samples using the MagMAX™-96 for Microarrays Total RNA Isolation Kit (Applied Biosystems, Waltham, MA, USA), while the MagMAX™ Pathogen RNA/DNA kit (Applied Biosystems) was used for viral RNA extraction from serum samples. Both kits were run on a MagMAX™ Express Magnetic Particle Processor (Applied Biosystems) following the manufacturer’s instructions. Next, 5 µL of extracted product was added to 20 µL of the AgPath-ID™ One step RT-PCR master mix (Applied Biosystems). The RT-qPCR reactions were performed on an ABI 7500 Fast instrument (Applied Biosystems) run in standard mode with the following conditions: 1 cycle at 45 °C for 10 min, followed by 1 cycle at 95 °C for 10 min, 40 cycles at 95 °C for 15 s, and 60 °C for 45 s. The forward primer sequence was 5′-TGCCTGGTATTCGTGGC-3′, the reverse primer sequence was 5′-TCATCCCATGTTCCAGAGT-3′, and the probe sequence was 5′-CCTCCGTCTCCGCGGCTTCTTTGG-3′. The limit of detection was determined with a pestivirus ultramer DNA oligo (Integrated DNA Technologies, Coralville, IA, USA), and Ct values greater than 35 were considered negative.

### 2.4. Indirect Enzyme-Linked Immunosorbent Assays (ELISAs)

Serum samples were tested for antibodies against E^rns^ and NS3 proteins using indirect ELISA assays previously described [12]. In brief, 96-well polystyrene ELISA plates (Thermo Fisher Scientific, Waltham, MA, USA) were coated with 100 μL of diluted APPV E^rns^ (0.33 μg/mL) or NS3 (0.21 μg/mL) recombinant proteins in phosphate-buffered saline (PBS) of pH 7.4 per well, sealed, and incubated at 4 °C with wet paper towel for humidity for 16 h. Then, plates were washed 5 times (350 μL/well) with PBS of pH 7.4 containing 0.1% Tween 20 (PBST) and blocked with 1% bovine-serum-albumin solution (BSA, Jackson ImmunoResearch, Inc., West Grove, PA, USA) at room temperature (22–25 °C) for 2 h. Coated plates were loaded with 100 μL/well of serum diluted 1:100 in PBS containing fetal bovine serum (FBS; Gibco^®^, Thermo Fisher Scientific) and incubated at 37 °C for 1 h. Plates were washed 5 times with PBST, followed by the addition of 1:25,000 peroxidase (HRP)-conjugated goat anti-pig IgG antibody (Bethyl Laboratories Inc., Montgomery, TX, USA) per well, and incubated at 37 °C for 1 h. After another washing step, tetramethylbenzidine-hydrogen peroxide (TMB) substrate solution (Surmodics IVD, Inc., Eden Prairie, MN, USA) was added (100 μL/well) and incubated at room temperature for 5 min. Reactions were stopped with 100 μL/well of stop solution (Surmodics IVD, Inc.), and OD at 450 nm was measured using an ELISA plate reader (EMax Plus Microplate Reader^®^ Molecular Devices, San Jose, CA, USA) operated with commercial software (Softmax Pro 7.0, Molecular Devices). The serum antibody response was expressed as the sample-to-positive (S/P) ratio:

$$S/P\;\mathrm{ratio}\;=\frac{\left(sample\;mean\;OD-negative\;control\;mean\;OD\right)}{\left(positive\;control\;mean\;OD-negative\;control\;OD\right)}$$

The cutoff for the NS3 ELISA was 0.4, and for the E^rns^ ELISA, the cutoff was 0.1.

### 2.5. In Situ Hybridization

Tissue sections were placed in 10% neutral-buffered formalin for 24 h, paraffin-embedded, and sectioned. An in situ hybridization (ISH) assay (RNAscope; Advanced Cell Diagnostics, Newark, CA, USA) was used for the detection of APPV viral RNA in pig tissues using the RNAscope 2.5 HD Reagents–RED kit (Advanced Cell Diagnostics) as previously described [16]. Proprietary ZZ probes targeting the 5′ UTR-N^pro^ coding region of the APPV strain sequenced from samples collected from pigs enrolled in the study were designed and manufactured by Advanced Cell Diagnostics. The APPV sequence was obtained from Sanger sequencing using the following primers: forward primer 5′-CTGAGAGAGAGGTACCGAACTCTTAAG-3′ and reverse primer 5′- TCACAATTGGGTTTCCATTGGTA-3′. A positive porcine control probe (SS-UBC) and a negative control probe (dapB universal) were utilized (Advanced Cell Diagnostics).

### 2.6. Data Analysis

Graphs were prepared using the GraphPad Prism Version 8.1.2 (332) software.

## 3. Results

### 3.1. Congenital Tremors

**Figure 1:**
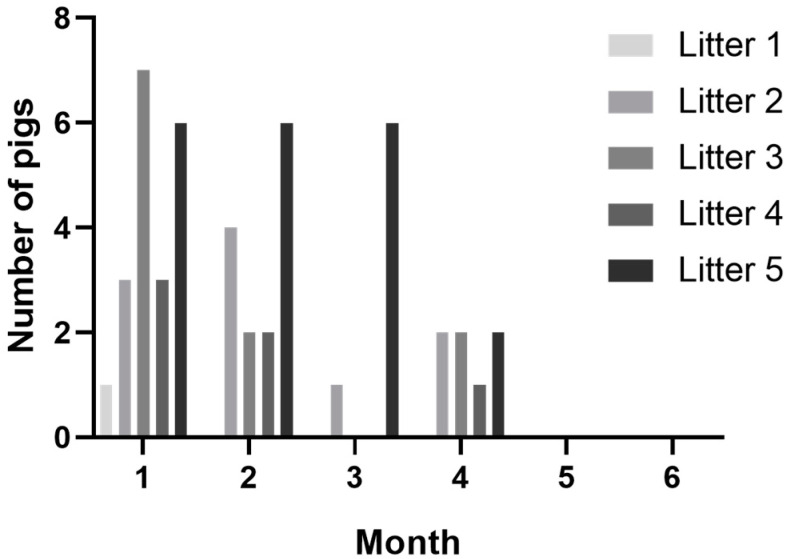
Number of pigs observed with tremors from CT-positive litters by month.

Twenty-five pigs out of the sixty (46%) pigs in CT-positive litters demonstrated tremors at one or more timepoints during observations. Most pigs were observed with tremors on the first sampling timepoint (20/60), and the number of pigs observed with tremors declined over each sample timepoint, with clinical signs resolving in all animals by the end of study (Figure 1). While a majority of pigs with CT were documented on the first timepoint, five pigs were observed with tremors for the first time at later timepoints. A little over half (14/25) of CT-positive pigs were recorded as having tremors on two or more observations. The percentage of affected pigs per litter ranged from 8–75% within CT-positive litters (Table 1). There were a similar number of male and female affected pigs. No pigs from CT-negative litters ever developed tremors, even after co-mingling at the wean-to-finish farm.

### 3.2. APPV RNA Detection by RT-qPCR

**Figure 2:**
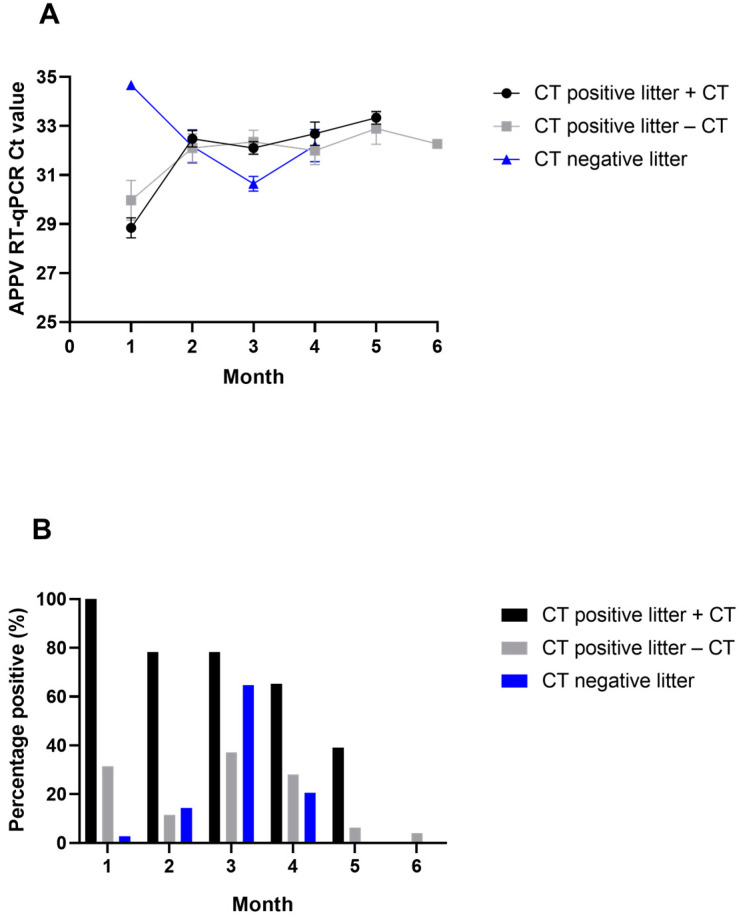
Detection of APPV RNA by RT-qPCR in serum. (**A**) Mean Ct value of positive serum samples by month. Error bars are standard error of the mean. (**B**) Percent of animals positive for APPV in serum by month. Positive samples had Ct values ≤ 35.

All pigs observed with tremors during the study (25/25) were PCR positive in serum at the first sample timepoint (Month 1); however, 11/35 (31%) of pigs without CT but born in CT litters were also PCR positive (Figure 2A). Only one pig born in a CT-negative litter had a PCR-positive serum sample at the first timepoint, with a Ct value of 34.7 (Figure 2B). The second sample timepoint (Month 2) was collected after pigs from CT-positive litters and CT-negative litters were co-mingled at the wean-to-finish barn. There was a decrease in the number of PCR-positive serum samples in pigs born in CT-positive litters, while the number of PCR-positive pigs began to increase for those born in CT-negative litters. By Month 3, the number of PCR-positive pigs born in CT-negative litters peaked (22/34, 65%), as well as the average quantity of APPV RNA detected in this group. By Month 5, viral RNA was no longer detected in serum from pigs born in CT-negative litters. Ten pigs from CT-negative litters never tested PCR positive in serum for APPV during the study.

Differences within CT-positive litters were observed when comparing pigs that demonstrated tremors compared with those that did not develop tremors. Even at the peak of viral detection among those pigs that did not develop tremors in CT-positive litters (Month 3), only 37% (13/35) of the pigs were PCR positive (Figure 2A). In contrast, 78% (18/23) of pigs that had CT were PCR positive at the Month 3 sampling. By Month 5, 39% (9/23) of CT-positive pigs were still PCR positive, while only 6% (2/32) of those that did not develop tremors were still positive. Almost half (43%) of pigs that did not develop tremors never had a PCR-positive result in serum. The average genomic copies for PCR-positive samples in both those pigs that had CT and those that did not were similar (Figure 2B).

### 3.3. APPV Antibody Detection by ELISA

**Figure 3:**
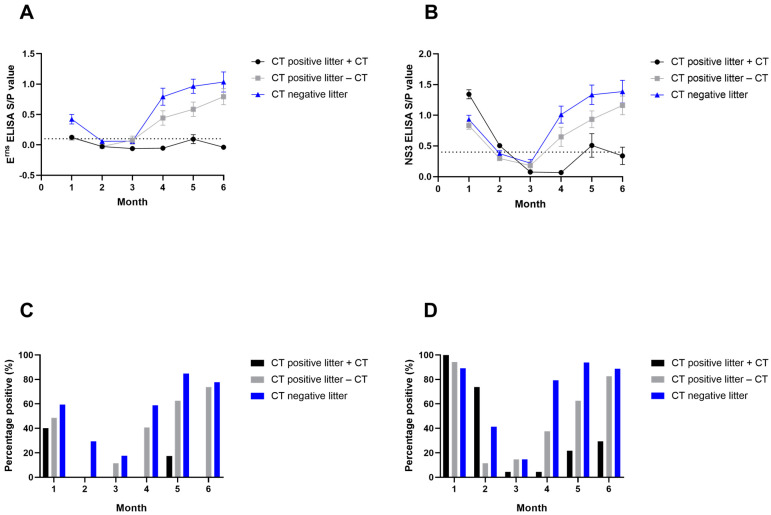
E^rns^ and NS3 ELISA results. (**A**) Mean S/P ratio value of the E^rns^ ELISA run on serum samples by month. Error bars are standard error of the mean (SEM). (**B**) Mean S/P ratio value of the NS3 ELISA run on serum samples by month. Error bars are SEM. (**C**) Percent of animals positive for the E^rns^ ELISA in serum by month. Positive samples had S/P ratio values ≥ 0.1. (**D**) Percent of animals positive for the NS3 ELISA in serum by month. Positive samples had S/P ratio values ≥ 0.4.

Evidence of maternal immunity was present in pigs from both CT-positive and CT-negative litters for both ELISAs; however, the NS3 ELISA detected a greater percentage of APPV-seropositive pigs compared with the E^rns^ ELISA. For the NS3 ELISA, 33/37 (89%) pigs born in CT-negative litters and 58/60 (97%) pigs in CT-positive litters were seropositive (Figure 3A). Additionally, results from the E^rns^ ELISA showed 22/37 (59%) seropositive pigs in CT-negative litters and 27/60 (45%) seropositive pigs from CT-positive litters at Month 1 (Figure 3B). CT-positive pigs had the highest average S/P ratio values for the NS3 ELISA in Month 1 (Figure 3C). In contrast, CT-negative litters had the highest average S/P values for the E^rns^ ELISA (Figure 3D). Among those pigs born in CT-positive litters with evidence of maternal immunity, all pigs with CT had APPV-PCR-positive serum at Month 1, while only 4/17 (24%) of those that did not develop tremors within CT-positive litters were PCR positive at that time.

There was a decrease in the percentage of seropositive pigs and S/P values for both ELISAs from Month 1 through Months 2 and 3 for most pigs, which provided evidence of waning maternal immunity (Figure 3). By Month 4, pigs from CT-negative litters, as well as pigs without CT but born in CT-positive litters, began to develop individual immune responses to APPV, with an increase in S/P values and the number of seropositive pigs; however, this increase was not observed for CT-positive pigs. Pigs that demonstrated tremors were less likely to develop an individual immune response, and the average S/P values were below or close to the cutoff after weaning. Pigs from CT-negative litters consistently had higher titers than those from CT-positive litters throughout the growing phase (Figure 3).

### 3.4. APPV RNA across Tissues

Almost six months after the animals were born, there was still evidence of viral RNA detected by PCR in multiple tissues collected from both groups of pigs with CT (n = 4) as well as those from CT-negative litters (n = 4) (Table 2). The quantity of APPV RNA was similar between groups in the tissues tested; however, some tissues had even lower Ct values than those detected in serum, including the cerebellum and nasal turbinates. Due to the possibility of vertical transmission of APPV from gilts/sows to offspring, sex organs of females (n = 3) were also tested for APPV RNA by PCR, and all tissues were negative. Cerebellum and nasal turbinates were the only tissues collected that were positive in all the animals tested.

**Figure 4:**
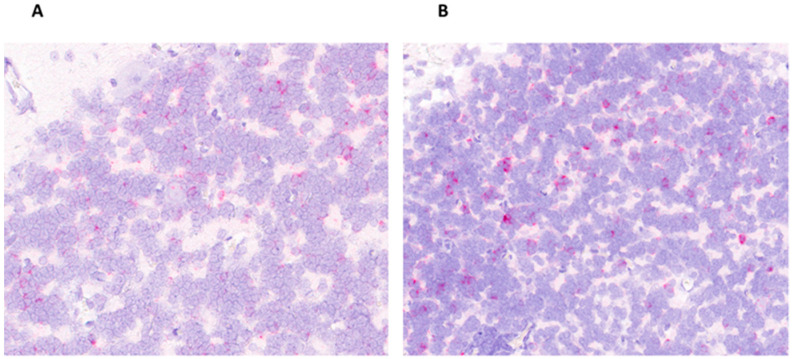
In situ hybridization detection of APPV in the cerebellum of both a pig with CT and a pig born in a CT-negative litter. (**A**) CT-positive pig (#55) cerebellum with multifocal-positive labeling (red dots) of APPV RNA in the granular layer. (**B**) CT-negative pig (#61) cerebellum with multifocal-positive labeling (red dots) of APPV RNA in the granular layer. Original magnification 200×.

The RNAscope demonstrated the presence of APPV in the cerebellum, confirming the comparable PCR results between pigs with CT and pigs born in CT-negative litters. Moreover, a similar multifocal-to-segmental distribution of viral RNA in the granular cell layer of the cerebellum was observed between the litter statuses (Figure 4). Viral RNA was not observed in the molecular layer, Purkinje cell layer, or medulla of the white matter.

## 4. Discussion

Atypical porcine pestivirus was discovered through sequencing efforts in 2015; however, gaps in our understanding of this pestivirus remain [5]. Although there have been recent advances, difficulties in isolating APPV in cell culture and working with the virus in vitro have slowed the progression of research efforts. To work around these limitations, APPV research has focused on field-based efforts to study the virus in vivo. The goal of this research was to gain a better understanding of the pathogenesis of APPV in swine by following a large cohort of pigs over time to characterize viral detection and the correlating humoral immune response specifically comparing CT-positive pigs with littermates without CT as well as those born in CT-negative litters.

There was a large variation in the number of pigs that developed tremors between the CT-positive litters, which is similar to that previously reported by both observational studies in the field and experimental challenge studies [1,4,7,14,24]. Even in experimental infections where each fetal amniotic vesicle was directly inoculated, not all pigs developed CT [4]. It is still unknown why some pigs develop tremors and others do not, as APPV has been detected in both symptomatic and asymptomatic pigs within affected litters [4,11,24,25]. Clinical disease is likely a combination of factors that could include timing of infection, quantity of viral exposure, innate immune response, and genetics. Not only can there be variation in the number of pigs that develop tremors in a litter but also the severity of tremors, which can range from slight muscle twitches to the entire body trembling [2,14]. Pre-weaning mortality can increase due to the severity in some pigs, but for most pigs, clinical signs will eventually resolve over time, as was observed in pigs followed during this study [7,24,26].

In this study, the number of PCR-positive pigs was much lower in pigs that did not develop tremors compared with those pigs that had CT within CT-positive litters. A majority of CT-positive pigs in this investigation were also still PCR positive four months into sampling; however, the number did decline by the end of the study. A similar viremia profile in CT- and APPV-positive pigs has been reported in other studies following smaller groups of animals [1,11,14,15]. Horizontal transmission was observed between pigs from CT-positive litters and those born in CT-negative litters during co-mingling after weaning, suggesting a productive infection and infectious virus present. In contrast to the persistence observed in pigs with CT, those pigs born in CT-negative litters became infected and were able to clear the virus from serum below a detectable level over a month or two. Similarly, Cagatay et al. [11] observed a more transient viremia and timely development of an antibody response in pigs (n = 5) from litters without CT after co-mingling with pigs from CT-positive litters (n = 15) during a longitudinal study. The present study supports the important role the timing of pestivirus infection plays in infection dynamics and immunocompetence, especially comparing animals infected by vertical transmission compared with horizontal transmission [19,27].

Almost half of pigs born in CT-positive litters did not develop tremors and never tested PCR positive for APPV in serum; however, most of these animals eventually developed an individual immune response, as observed by seroconversion. In contrast, only ten pigs born in CT-negative litters (27%) did not test PCR positive for APPV. Due to APPV-positive pigs in all CT-positive litters and evidence of transmission to pigs born in CT-negative litters co-mingled with CT-positive litter pigs at weaning, it is likely that most animals in this study were exposed to APPV at some point during the study. Perhaps exposure occurred when protective maternal antibodies were present or they were able to mount an individual immune response that was effective in clearing the virus to an undetectable level by the time that sampling had occurred. Further research including more frequent sampling could help characterize differences in viral infection dynamics observed between pigs with CT and those that do not but are born in the same litter and would be beneficial to better understanding APPV immunopathogenesis.

The timing of pestivirus infection and transmission from dams to their offspring plays a significant role in outcomes observed for those offspring. Depending on the stage of gestation, transplacental infection of BVDV or CSFV can result in absorption, persistently infected animals, abortion, malformations, or a normal immunocompetent animal [19,27]. Clinical signs of persistently infected animals can vary from unthrifty to apparently healthy, but they are immunotolerant to the infecting strain and remain viremic for life [19,27]. There is no way to identify when the CT-positive litters were exposed in utero to APPV in this study, but evidence would suggest that these pigs did not fit the classic characteristics of persistently infected animals listed above. By the last serum sample, all CT-positive pigs were PCR negative for APPV, and some of these animals had S/P values above the cutoff, indicating the development of an individual immune response. Therefore, further research could improve our understanding of the delay in immune response and viral clearance from serum and tissues.

All pig serum samples were PCR negative for the CT-negative litters and the CT-positive pigs by Months 5 and 6, respectively; however, multiple tissues from pigs of both groups were positive for APPV RNA at the time of marketing. Some tissue samples had low Ct values compared with that observed in the serum, especially the cerebellum, which interestingly did not have any obvious pathologic changes. Further work needs to be conducted to determine if the virus enters immune-privileged sites and is protected and persists. Positive salivary-gland and nasal-turbinate tissues could suggest these animals may be able to transmit the virus in nasal or oral secretions after the resolution of clinical signs and clearance of viremia. Other research has also reported the detection of APPV in oral swabs of animals that had demonstrated CT at six months of age and tissues from CT-recovered boars at 11 months of age [14,16]. In addition, cotton ropes hung in pens housing the experimental animals in this study were PCR positive near the time of marketing. Thus, oral fluids may be a convenient antemortem sample specimen to collect to monitor for APPV, while multiple tissues could be collected postmortem to check for APPV status.

In this study, there was evidence of maternal immunity across both CT-positive and CT-negative litters; however, there was more evidence of maternal immunity using the NS3 ELISA compared with the E^rns^ ELISA. Interestingly, approximately half of the pigs in CT-positive litters with evidence of maternally derived antibodies were also PCR positive for APPV in the serum, which could be explained by previous research demonstrating maternally derived antibodies that only displayed low-to-moderate neutralizing capacity [11]. Maternal antibodies may not protect well against infection and replication of APPV. In addition, the assessment of antibody levels by ELISA may not be the best measurement for protective immunity. Pigs sampled at Month 1 varied in age from three days to approximately two weeks of age; therefore, the timing of infection was unknown. It is possible that APPV-positive piglets were infected in utero or after birth. In addition, it is unknown whether there were multiple strains of APPV circulating on this farm. After maternal antibody waned, pigs born in CT-negative litters developed an individual immune response as well as many of those pigs born in CT-positive litters but did not develop tremors. Again, few of the CT-positive pigs developed a detectable antibody response during the six months of sampling.

This study has expanded on previously published information on APPV viral dynamics in a field setting. Differences in the timing and/or amount of viral exposure during the gestation period may play a role in differences observed in APPV infection and immune dynamics. APPV–CT-positive pigs tended to have a longer viremia and a delayed or absence of detectable antibody responses; however, there was PCR evidence of viral persistence in tissues of all animals exposed to APPV. The presence of viral RNA at low Ct values in tissue suggests that persistently infected animals may be infectious and able to transmit the virus for extended periods of time and perhaps females could give birth to CT piglets. The somewhat delayed (approximately 2 months) immune response in a majority of animals from non-CT litters should inform exposure protocols in the absence of available, efficacious vaccines. Further research into the ability of the virus to evade the immune response, the duration and primary route of shedding by infected animals, and vaccine candidates will be important to formulate control measures to reduce the impact of APPV in the swine industry.

## Figures and Tables

**Table 1:** Congenital tremor observations by litter.

Dam #	Parity	Pig Age When Enrolled (Days)	Litter Size	CT-Positive Pigs (%)	# Males (# CT-Positive Males)	# Females (# CT-Positive Females)
1	1	13	12	1 (8%)	7 (0)	5 (1)
2	1	13	11 *	4 (36%)	4 (1)	6 (2)
3	1	13	12	8 (67%)	5 (3)	7 (5)
4	1	13	13	3 (23%)	10 (3)	3 (0)
5	3	3	12 *	9 (75%)	6 (4)	5 (4)
6	1	17	11	0 (0%)	7 (0)	4 (0)
7	1	16	13 *	0 (0%)	3 (0)	9 (0)
8	3	4	13 ^#^	0 (0%)	6 (0)	5 (0)

* Sex not recorded on one pig. ^#^ Sex not recorded on two pigs.

**Table 2:** PCR Ct values of selected tissue samples.

Pig # (Sex)	CT Status of Litter	Right Cerebellum	Left Cerebellum	Nasal Turbinate	Mandibular Lymph Node	Mandibular Salivary Gland	Tonsil	Palatoglossal Arch
50 (F)	Positive	21.8	20.9	27.0	ND	26.6	32.3	29.5
52 (M)	Positive	21.9	22.3	29.1	ND	32.5	ND	29.8
53 (M)	Positive	24.9	24.0	26.0	ND	ND	ND	ND
55 (M)	Positive	19.4	20.2	22.5	32.9	26.1	29.8	32.3
61 (M)	Negative	21.5	21.6	24.7	ND	25.5	28.3	28.1
62 (F)	Negative	24.0	34.0	23.7	ND	33.5	33.9	33.4
70 (F)	Negative	30.9	24.3	21.2	ND	29.8	31.8	32.0
91 (M)	Negative	34.9	20.0	22.5	ND	ND	ND	ND

ND = Not determined.

## Data Availability

The data presented in this study are available on request from the corresponding author.
